# Associations between a Subjective Living Environment and Quality of Life among People with Arterial Hypertension—Results from the Hamburg City Health Study

**DOI:** 10.3390/ijerph20010180

**Published:** 2022-12-22

**Authors:** Jobst Augustin, Ramona Bei der Kellen, Christian-Alexander Behrendt, Christina Magnussen, Claudia Terschüren, Leonie Ascone, Simone Kühn, Sandra Wolf, Matthias Augustin, Valerie Andrees

**Affiliations:** 1 Institute for Health Services Research in Dermatology and Nursing (IVDP), University Medical Center Hamburg-Eppendorf (UKE), 20251 Hamburg, Germany; 2 Epidemiological Study Center, Hamburg City Health Study (HCHS), University Medical Center Hamburg-Eppendorf (UKE), 20251 Hamburg, Germany; 3 Department of Cardiology, University Heart & Vascular Center Hamburg, University Medical Center Hamburg-Eppendorf (UKE), 20251 Hamburg, Germany; 4 Population Health Research Department, University Heart & Vascular Center Hamburg, 20251 Hamburg, Germany; 5 German Centre for Cardiovascular Research (DZHK), DZHK-Geschäftsstelle, 10785 Berlin, Germany; 6 Institute for Occupational and Maritime Medicine (ZfAM), University Medical Center Hamburg-Eppendorf (UKE), 20251 Hamburg, Germany; 7 Clinic and Polyclinic for Psychiatry and Psychotherapy, University Medical Center Hamburg-Eppendorf (UKE), 20251 Hamburg, Germany; 8 Lise Meitner Group for Environmental Neuroscience, Max Planck Institute for Human Development, 14195 Berlin, Germany

**Keywords:** Hamburg City Health Study, arterial hypertension, subjective living environment, quality of life, city

## Abstract

Hypertension is a global public health concern and an important contributor to cardiovascular disease. It remains disputed how important life circumstances are for the etiology of hypertension. Thus, the aim of this study is to assess the spatial variation of hypertension within an urban population and to investigate the association with the quality of life of city dwellers and their subjective evaluation of their residential district, as well as their home environment, using the example of Hamburg, Germany. In this cross-sectional study, the first 10,000 participants from the Hamburg City Health Study (HCHS) were analysed. Only participants who had resided at the current address for a minimum of five years were considered. In the descriptive analysis, participants with and without arterial hypertension were compared considering various parameters. The subjective quality of the living environment was obtained using an appropriate subjective living environment index. Quality of life was mapped using the EuroQol Group quality of life questionnaire (EQ-5D) score and the two (mental and physical health) scores of the Short Form Health Questionnaire SF-8. The Gini-coefficient was used to quantify the regional economic variation within Hamburg. Linear and logistic regression analyses were performed. Regional levels were 68 city district clusters in Hamburg. The analysis included n = 8192 participants living at least five years in Hamburg at the time of participation in the HCHS. There was a spatial variation in the prevalence of arterial hypertension within Hamburg. Prevalence rates between city district clusters ranged from 50.0% to 88.5%. The results showed that city district clusters with a worse subjective perception of the living environment were partly associated with an increased prevalence of arterial hypertension. Furthermore, a negative association was observed between arterial hypertension prevalence and the sociodemographic status of participants in the city district clusters. Thus, participants with a high level of education suffered less frequently from arterial hypertension than participants with a rather low level of education. The subjective living environment index and quality of life were significantly related to the occurrence of arterial hypertension; however, more extensive and detailed studies are necessary to derive possible clinical implications.

## 1. Introduction

Hypertension is a significant world-wide public health concern [1]. Hypertension affects more than 1.2 billion people worldwide and is associated with approximately 8 million premature deaths per year. Against the background of an ageing population, this impact will become even more pronounced [2]. This trend has also been observed in recent decades in low- and middle-income countries [3]. The most prevalent form of hypertension is arterial hypertension, i.e., elevated systemic blood pressure. With increasing frequency and high direct and indirect consequential costs, it represents a particular burden for the public health system [4]. The disease is considered one of the main risk factors for morbidity and mortality from other diseases worldwide and is particularly associated with the occurrence of cardiovascular diseases (e.g., coronary heart disease and peripheral arterial occlusive disease) [5]. It emerges from various factors, which include, above all, the interaction of hereditary factors, age, sex, and unfavourable nutritional (e.g., salt and alcohol consumption) and living conditions (e.g., lack of opportunities for physical activity), as well as the living environment [4,6,7]. With regard to the living environment, the focus is on urban areas, as the importance of arterial hypertension-associated determinants is substantially high here. These include air quality [8], noise [9], light pollution [10], green spaces with opportunities for outdoor physical activity [11,12], settlement density, perceived safety [13], and access to healthy food [14]. 

In an international comparison, Germany records the highest prevalence of arterial hypertension [15]. According to the results of the GEDA (Gesundheit in Deutschland aktuell) 2014/2015-EHIS (European Health Interview Survey) study involving 24,016 participants, almost one in three adults (30.9% of women, 32.8% of men) aged 18 years and older had medically diagnosed arterial hypertension. However, the prevalence rates are subject to regional variations [7,16,17]. In an east-west comparison, prevalence is higher in the eastern federal states of Germany, likely as a result of different regional, sociodemographic conditions. Neuhauser et al. identified an association of prevalence rates with educational status [7]. For example, higher-educated women significantly less often have arterial hypertension than women in the lower educational group. In men, this association was only observed in the age group of 45 to 64 years. The importance of socio-economic status, especially educational status, has also been demonstrated and documented by Cuschieri et al. and Krefis et al. in 2017 [18,19]. Holstiege and his colleagues (2020) revealed that the extent of regional deprivation of the county of residence is related to the risk of arterial hypertension [16]. For example, among 20–49-year-olds, the risk was increased by a factor of two in eastern German regions with the highest deprivation compared to western German regions with the lowest deprivation. Kauhl et al. (2018) analysed the spatial distribution of arterial hypertension at the municipality-level to assess location-specific associations between arterial hypertension, sociodemographic population characteristics, and area deprivation in northeastern Germany [20]. The main risk factors for arterial hypertension involved mean age, area deprivation, and proportion of people commuting to work outside their home municipality.

Another small-scale study was conducted by Faka et al., which investigated the influence of regional socioeconomic and environmental factors on arterial hypertension [21]. Here, associations were identified between higher disease rates with higher social inequality and poorer environmental conditions. 

However, a shortcoming of many studies is that they are not spatially small enough and often do not permit statements on spatial variations in a more ‘controlled’ context, e.g., such as in the same city. Therefore, the assessment of the inner-city situation is of great importance because striking spatial variations often occur there, and targeted intervention measures are required. Lack of data in Germany is often the reason for this. Furthermore, most of the studies do not include a sufficiently large number of cases to produce valid results. In addition, they often have a limited set of variables to adjust the statistical models in order to make more differentiated statements about specific aspects of the overall living environment, the associated quality of life, and the risk of arterial hypertension. The aim of this study is, therefore, to show the spatial variation of arterial hypertension within the city of Hamburg. Moreover, we want to analyse the association with the quality of life of city dwellers and their subjective evaluation of their home, as well as their residential district living environment. 

## 2. Methodology 

### 2.1. Study Design and Population

These cross-sectional analyses are based on the first 10,000 participants of the Hamburg City Health Study (HCHS; NCT03934957), a regional cohort study of Hamburg residents aged 45–74 years. They are randomly selected by the residents’ registration office [22]. The city of Hamburg is, with 1.9 million inhabitants, the second largest city in Germany. As in other cities, a pronounced social gradient [23] can be found in the urban population of Hamburg. 

### 2.2. Data Collection

The recruitment of the HCHS cohort (first wave) took place between April 2016 and November 2018. As a reference to the place of residence is established in this study, only those participants were considered who indicated a minimum duration of residence of five years at the current residential address. The duration of residence of the participants was surveyed in the HCHS by means of self-report. The daily time spent at home (in hours) was assessed as a second home environment covariate. The main questionnaire contained variables for a more objective description of the home environment (e.g., type and qualities of the building/home, such as type of heating), and also assessed the subjective evaluation of the home (e.g., satisfaction with protection from disturbing night light), as well as the residential district environment (e.g., subjective safety of district). We used variables containing the subjective evaluation of the home, as well as the residential district environment, to build a subjective living environment index. 

Arterial hypertension is recorded in the HCHS in three different ways: (1) arterial hypertension is assumed if the respondent answers in the affirmative to the question “Do you have a doctor-diagnosed hypertension?”, (2) in addition, arterial hypertension is assumed if antihypertensive medication has been taken (with ATC code groups C09A, C09C, C07A, C03C, C03A, C03D, C08C, C02D, C02A, C09X or C01D), or if (3) a systolic blood pressure of over 140 mmHg or a diastolic blood pressure of over 90 mmHg at rest has been measured [3].

The subjective living environment index included the items: disturbance by traffic, and attractiveness of the residential district, shopping facilities nearby, social contacts in the neighbourhood, ambient air quality, green zones nearby, and safety of the neighbourhood (as subjective evaluation of the residential district environment). The respondents evaluated the characteristics of their residential district environment on a scale ranging from 0–4 (0 = very poor; 1 = rather poor; 2 = partly; 3 = rather good; 4 = very good). The latter seven variables were summed into a subjective living environment index for the purposes of this analysis. Scores, therefore, ranged from 0 to 28. Higher scores indicate a more positive subjective evaluation. 

In addition, the subjective evaluation of the home environment was taken into account in the analysis (e.g., brightness of the rooms, disturbance at home by noise), as well as the subjective evaluation of the residential district environment (e.g., shopping facilities nearby, social contacts in the neighbourhood). Educational status was classified according to ISCED (International Standard Classification of Education [24]) as low (ISCED = 1), medium (ISCED = 2), and high (ISCED = 3). Using this model, prevalence of arterial hypertension was estimated per district cluster for a given value (age = 63 years, sex = male, educational status = medium, smoking = no, heart disease = none) of the variables to be adjusted for and visualised in a map. District clusters with less than 10 cases were excluded.

Quality of life was assessed with the score of the EurQol Quality of Life Questionnaire (EQ-5D) with five dimensions: mobility, self-care, usual activities, pain/discomfort, and anxiety/depression [25]. The score covers a range of values from −0.661 (worst possible state) to 1 (best possible state). Additionally, quality of life was assessed with the two scores of the Short Form Health Questionnaire SF-8 [26] (MS8 mental scale and PS8 physical scale). The SF-8 covers a range of values from 0 to 100. All variables included in the analyses are summarised in Table 1. For location-specific analyses, the 103 districts of Hamburg were grouped into 68 district clusters, according to the morbidity atlas [23].

### 2.3. Statistical Analysis

First, a descriptive analysis of the participants’ characteristic was conducted. For this, participants with and without arterial hypertension were compared by demographics, quality of life, subjective evaluation of home, residential district environment, and cardiovascular risk factors. These risk factors comprised smoking status, diabetes, peripheral arterial occlusive disease (PAOD), and other heart diseases. Those heart diseases included atrial fibrillation, acute coronary syndrome, coronary heart disease, angina pectoris, heart valve defect, myocarditis, and endokarditis.

Continuous variables are presented with means and standard deviations and categorical variables with absolute numbers and percentages. Missing values were excluded when calculating percentages and for further analyses. Mann–Whitney U tests for the continuous variables and chi-squared tests for the categorical variables were performed to detect significant differences between the two groups. A *p*-value of less than 0.05 was considered statistically significant. 

To quantify the regional variation, we used the Gini coefficient as a further statistical measure. The Gini coefficient measures the inequality between the values of a frequency distribution (here, prevalence between the city district cluster). A Gini coefficient of 0 is an expression of complete homogeneity between the city districts clusters, meaning that all prevalence rates are equal. A Gini coefficient of 1 describes the maximum inequality between the city district clusters [27].

In order to determine any spatial differences in the prevalence of arterial hypertension, a logistic regression analysis was carried out to describe the association between the district clusters of participants’ residence and the presence of arterial hypertension. This was adjusted for age, sex, educational status, smoking status (smoker or non-smoker), and heart diseases to control for the influence of these confounders. Maps were produced with the adjusted prevalence rates, as well as scores of the subjective living environment index, to display the differences graphically.

The association between arterial hypertension, quality of life, and the subjective living environment index was investigated using two models: (1) A logistic regression with arterial hypertension as outcome variable and (2) quality of life, as well as the subjective living environment index as determinants, was conducted. The odds ratio and respective confidence interval of the individual determinants, as well as the *p*-value, were tabulated. In addition, a linear regression model with quality of life as the outcome variable and the subjective living environment index, as well as arterial hypertension, as determinants was calculated. The estimated model coefficients (beta), including confidence interval of the individual determinants, as well as the *p*-value, were tabulated. The models were adjusted for age, sex, educational status, smoking status, and heart diseases. 

Data analysis was performed with R version 3.5.1 (R Foundation, Vienna, Austria) and cartographic visualisation with QGIS version 3.10.4 (QGIS Development Team, Cologne, Germany).

## 3. Results

A total of N = 8192 participants (probands with information about arterial hypertension and a living history of more than five years at the same place) were included in the study (51.3% female). Of these, 66.2% (n = 5422) had arterial hypertension. The mean age of the participants with arterial hypertension was 64.5 years and of the non-hypertensives 59.0 years. Among participants with high educational status, the proportion of participants without arterial hypertension (49.0%) was higher than those with arterial hypertension (41.3%). For medium and low educational status, the ratio was reversed, i.e., the proportion of participants with arterial hypertension was higher (Table 2). In terms of quality of life, the total score was almost the same for hypertensives and non-hypertensives. In the SF-8, there were significant, but very small, differences in the two scores mental and physical. Hypertensive participants suffered more often from diabetes and other previous illnesses (especially heart diseases) (Table 2) and spent more time at home during the week.

**Figure 1:**
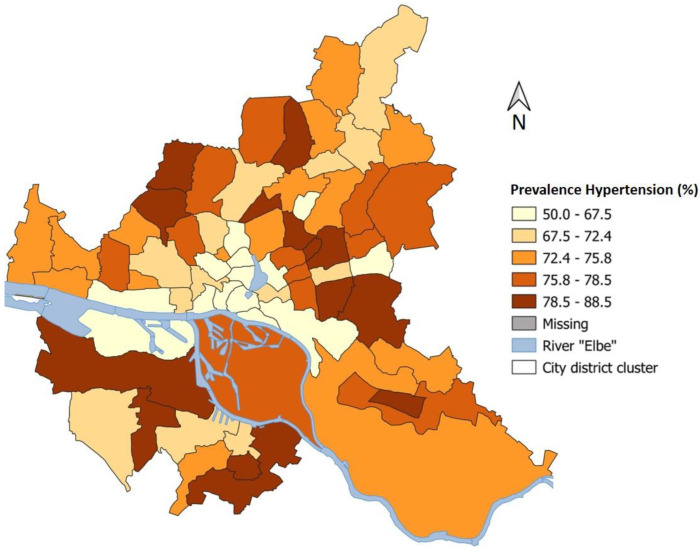
Prevalence of arterial hypertension (%) based on district clusters in Hamburg, adjusted for age, sex, educational status, smoking, and heart disease. Participants with at least five years of residence were considered.

Figure 1 shows the spatial distribution of arterial hypertension prevalence rates based on the 68 Hamburg district clusters. The underlying values were adjusted for age, sex, educational status, smoking, and heart disease. Relatively low prevalence rates (min. 53.2%) are found in the center of the city, while higher prevalence rates, with up to 88.5% hypertensives per district cluster, are found in the peripheral districts. This is particularly true for district clusters south of the river Elbe. The Gini coefficient for describing regional variation is 0.05.

**Figure 2:**
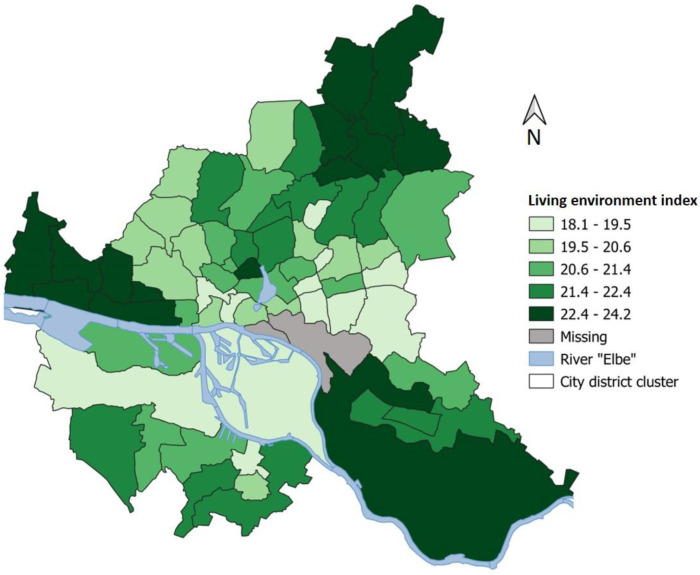
Scores of the subjective living environment index based on district clusters in Hamburg. Only participants with at least five years of residence were taken into account.

In Figure 2, the spatial characteristics of the subjective living environment index at the district cluster level are presented. The score ranges between 18.1 and 24.2 in the city district clusters. On average, a more positive subjective evaluation can be found in the north of Hamburg, as well as northwest of the Elbe and in the southeast of the city. The central district clusters tend to score lower on the subjective living environment index. The Gini coefficient is 0.04.

Scores of the subjective living environment index (total) differs only slightly between hypertensives (21.0 [95% CI 19.0, 24.0]) and non-hypertensives (22.0 [95% CI 19.0, 24.0]). In order to identify potential associations on a more fine-graded level, variables were also considered as separate indicators and not as a summary score. A distinction was made between the subjective evaluation of the home environment (Table 3) and the subjective evaluation of the residential district environment (Table 4). There were small differences with regard to variables of the subjective evaluation of the home environment. Hyperintensives feel less disturbed by noise during the day and at night. Of the hypertensives, 76.9% state that they are not disturbed by noise at night, compared to 72.9% of the non-hypertensives.

The subjective evaluation of the residential district environment (Table 4) differs slightly between hypertensives and non-hypertensives, with differences in the attractiveness of the residential district and the safety of the neighbourhood. Of the non-hypertensives, 39.0% rated their residential district as attractive, compared to 33.4% of the hypertensives. Among the non-hypertensives, 19.6% classified the safety of the neighbourhood as very good, compared to 16.4% among the hypertensives.

In order to quantify the relationships between arterial hypertension, quality of life, and the subjective living environment index, a logistic and a linear regression analysis (Table 5) were carried out. 

The logistic regression analysis, with arterial hypertension as the target variable, showed significant associations with quality of life (EQ-5D), age, gender, educational status (ISCED = 3), and previous illnesses (heart diseases). For example, the probability of developing arterial hypertension increases by 7.4% when age increases by one year. With regard to educational status, a high educational status (ISCED = 3) is associated with a 56.6% lower probability of developing arterial hypertension, compared to a low educational status (ISCED = 1). 

The linear regression analysis with quality of life measured by EQ-5D as the dependent variable (Table 5) highlights, among other things, significant associations with the subjective living environment index, arterial hypertension, gender, educational status (ISCED = 3), or heart diseases. If the subjective living environment index improves by one unit, it is associated with an increase in quality of life of 0.005 on the EQ-5D score. 

## 4. Conclusions

The aim of this study was to show the spatial variation of arterial hypertension within a city population and the association with the quality of life and the subjective evaluation of home and residential district environment using the example of Hamburg. 

The results illustrate a correlation between arterial hypertension prevalence and the sociodemographic status of the participants. Among the participants with a high educational status (according to ISCED classification), the proportion of hypertensives was lower than among the participants with a lower educational status. Regarding quality of life, measured with the EQ-5D or SF-8, there was only a small, but statistically significant, difference between hypertensives and non-hypertensives.

Individual risk factors were also evaluated. Here, it became apparent that the hypertensives suffer more frequently from diabetes and other comorbidities (especially heart diseases). In the regional evaluation, strong differences in prevalence were found. These ranged from 50.0% to 88.5% in the different city district clusters. The high prevalence rates in the district clusters south and northeast of the Elbe river are accompanied by a low subjective living environment index. In addition, on a single variable level, non-hypertensives tend to evaluate their residential district environment as significantly more attractive and safer than the hypertensives. To quantify the regional variations, Gini coefficients were calculated. With 0.05 (arterial hypertension prevalence) and 0.04 (living environment), the regional variations within Hamburg are small. A reason for the low variation may be that the distribution of study participants within Hamburg is uneven, and this is indirectly reflected in the regional variation measured here. There is a tendency towards less participation of people from socially weaker parts of the city in the HCHS.

The regression analyses indicate significant correlations of arterial hypertension prevalence with sociodemographic (age, educational status) and individual (previous illnesses) circumstances, as well as with the subjective living environment index and quality of life. Although many of the linear regression results were significant, the R^2^ of 0.07 is rather low. Therefore, the respective regression model can only predict a small part of the differences between hypertensives and non-hypertensives.

The results of this study are in accordance with other studies, which have examined only individual determinants (e.g., arterial hypertension and quality of life). In particular, the relationship of arterial hypertension with educational status could be proven by numerous studies; higher education is often associated with lower risk of arterial hypertension [7,18,19]. Here, we found that high education status (ISCED = 3) is associated with only about half the risk of hypertension compared to low educational status (ISCED = 1). Holstiege et al. also identified an association with social deprivation [16], and Faka et al. with other environmental determinants at the regional level [21]. The present study also suggests these associations, as we found attractiveness and safety of the living environment to be rated significantly different between hypertensives and non-hypertensives. The urban district clusters described by Erhart et al. as socially weak [23], for example in the south of Hamburg, likewise showed increased prevalence rates. Sociodemographic status is closely related to the home and residential district area environment, which, in its subjective evaluation, is reflected in the prevalence of arterial hypertension in our study. The added value of a spatial analysis becomes clear because the results illustrate the correlations between the frequency of arterial hypertension, the social situation, and, related to this, the overall subjective evaluation of the home and residential district environment on a small scale.

However, there are uncertainties and limitations of this study. In this study, the proportion of smokers among hypertensives is lower than among non-hypertensives in the HCHS, which is not consistent with other study results [28]. The relationship between arterial hypertension and disturbance by noise is also not clear-cut. Hypertensives (72.9%) tended to feel less disturbed by noise during the day and at night than non-hypertensives (76.9%). Although the difference is not too big, it contrasts with other study results that indicate that disturbance by noise at night is an important determinant of arterial hypertension [29]. One reason for this could be that the data used in this study are self-reported and, hence, pose bias. This is likely to be the case, especially in the case of disturbance by noise, or also in the assessment of perceived safety of the neighbourhood. Another explanation is that the index, as used in our study, is not optimal. Future studies could focus on developing and validating (including factorization) an exhaustive self-report assessment to derive an index of home and residential district subjective quality. In addition to subjectivity as a limiting factor, it must also be assumed that the HCHS study is subject to a selection bias in participation according to age, place of residence, or social status. These limitations could be contributing factors for the very high prevalence rates in some districts or district clusters. Within the HCHS, only cross-sectional data have been collected thus far; hence, associations based on a longitudinal view are not yet possible. 

Overall, the study was able to show associations between the subjective evaluation of the home and residential district environment, quality of life, and prevalence of arterial hypertension using the example of Hamburg. Thus, this study complements the results of other studies, whose validity is limited due to small populations. Data from the HCHS were used for the first time for this purpose. A strength of this study is the large sample size with more than 8000 participants, allowing for a certain degree of significance. In addition, the extensive questionnaires and examinations of the HCHS enable more detailed analyses than previous studies. 

In the future, small-scale and objectively assessed environmental data (e.g., noise levels, air pollutants, proportions of and access to green spaces) should be applied to develop an objective measure. This can be used as a complement and comparison to, as well as validation of, the subjective data of the participants. In addition, in the future, it will be possible to analyse the data longitudinally such that changes in and directions of associations can be detected. Moreover, more attention should also be paid to a possible bias due to different participation rates in the districts with different sociodemographic compositions. A correction factor can possibly be developed and taken into account in the future. The results of the study are particularly important from a public health perspective and once again illustrate the relevance of a small-scale approach to health. Intervention measures to improve population health can be used in a more targeted way.

## Figures and Tables

**Table 1:** Overview of used data in this study.

Name	Description	Variables	Entity
Demography	Age		Years (median IQR)
	Sex	Male Female	n (%)
	Family status	Married, cohabiting Married, living separately Single Divorced Widowed	n (%)
	Education ^1^	Low Middle High	n (%)
	Employment	Ours per week Unemployed	n (%)
Quality of life	EQ-5D		Score (median IQR)
	SF-8, psychical		Score (median IQR)
	SF-8, physical		Score (median IQR)
Risk factors	Smoking	Yes	n (%)
	Diabetes		
	Heart diseases ^2^		
	PAOD		
Home environment covariates	Type of house	Apartment house	n (%)
	Daily time spent at home (hrs)	During the week	hours (median IQR)
Subjective evaluation of home environment	Disturbance by noise, during the day in the week	Not at all A little Quite a lot Very	n (%)
	Disturbance by noise, during the day at the weekend		
	Disturbance by noise, night		
	Brightness of the rooms during the day		
	Disturbance by traffic *		
Subjective evaluation of residential district environment	Attractiveness of the residential district *	Very poor Poor Moderate Rather good Very good	n (%)
	Shopping facilities nearby *		
	Social contacts in the neighbourhood *		
	Ambient air quality *		
	Green zones nearby *		
	Safety of the neighbourhood *		

IQR: interquartile range, EQ-5D: EurQol quality of life questionnaire, SF-8: Medical Outcomes Survey Short Form-8 questionnaire, PAOD: peripheral arterial occlusive disease. * Variables used for the subjective living environment index. ^1^ Unesco (2012) International Standard Classification of Education (ISCED). ^2^ History of atrial fibrillation, acute coronary syndrome, coronary heart disease, angina pectoris, heart valve defect, myocarditis, endokarditis.

**Table 2:** Description of the study population.

Study Population		Total N = 8192	Without Hypertension n = 2770	Hypertension n = 5422	Test for Differences in Groups	*p*-Value
Demography						
Age	Years, median (IQR)	63 (56–70)	58 (52–65)	66 (58–71)	Mann–Whitney U test	<0.001
Sex	Male, n (%)	3390 (49.7)	1074 (38.8)	2916 (53.8)	Chi-squared test	<0.001
	Female, n (%)	4202 (51.3)	1696 (61.2)	2506 (46.2)	Chi-squared test	<0.001
Family status	Married, cohabiting, n (%)	5380 (66.6)	1738 (63.7)	3642 (68.1)	Chi-squared test	<0.001
	Married, living separately, n (%)	162 (2.0)	56 (2.1)	106 (2.0)		
	Single, n (%)	1009 (12.5)	431 (15.8)	578 (10.8)		
	Divorced, n (%)	1019 (12.6)	366 (13.4)	653 (12.2)		
	Widowed, n (%)	507 (6.3)	137 (5.0)	370 (6.9)		
Education	Low, n (%)	374 (4.6)	88 (3.2)	286 (5.3)	Chi-squared test	<0.001
	Middle, n (%)	4163 (51.5)	1307 (47.8)	2856 (53.4)		
	High, n (%)	3546 (43.9)	1338 (49.0)	2208 (41.3)		
Employment	Hours per week, median (IQR)	38.0 (29.0–40.0)	38.0 (28.0–40.0)	39.0 (30.0–40.0)	Mann–Whitney U test	0.053
	Unemployed, n (%)	169 (4.9)	76 (5.8)	93 (4.4)	Chi-squared test	0.086
Quality of life						
EQ-5D	Score, median (IQR)	0.94 (0.89–1.00)	0.94 (0.91–1.00)	0.94 (0.89–1.00)	Mann–Whitney U test	<0.001
SF-8, psychical	Mental Component Score, median (IQR)	57.25 (51.57–58.58)	57.05 (50.57–58.45)	57.25 (52.08–58.64)	Mann–Whitney U test	<0.001
SF-8, physical	Physical Component Score, median (IQR)	52.57 (46.43–56.35)	53.55 (48.07–56.68)	52.15 (45.29–55.80)	Mann–Whitney U test	<0.001
Risk factors						
Smoking	Yes, n (%)	1572 (19.3)	623 (22.5)	949 (17.6)	Chi-squared test	<0.001
Diabetes	Yes, n (%)	648 (8.4)	69 (2.6)	579 (11.4)	Chi-squared test	<0.001
Heart diseases	Yes, n (%)	1383 (20.3)	186 (8.0)	1197 (26.6)	Chi-squared test	<0.001
Self-reported PAOD	Yes, n (%)	278 (3.5)	66 (2.5)	212 (4.1)	Chi-squared test	<0.001

N: number of participants, n: number of persons, IQR: interquartile range, EQ-5D: EurQol quality of life questionnaire, SF-8: Medical Outcomes Survey Short Form-8 questionnaire, PAOD: peripheral arterial occlusive disease. *p*-value of significance tests for the difference between the groups.

**Table 3:** Home environment covariates and subjective evaluation of home environment.

		Total N = 8192	Without Hypertension n = 2770	Hypertension n = 5422	*p*-Value
Home Environment Covariates					
Type of house	Apartment house, n (%)	4556 (56.0)	1522 (55.1)	3034 (56.5)	0.233 ^1^
Daily time at home in hours	During the week, median (IQR)	14.0 (12.0–18.0)	14.0 (12.0–16.0)	15.0 (12.0–18.0)	<0.001 ^1^
	Weekends, median (IQR)	16.0 (14.0–19.0)	16.0 (14.0–19.0)	16.0 (14.0–20.0)	0.322 ^1^
Subjective evaluation of home environment					
Disturbance by noise, during the day in the week	Not at all, n (%)	4726 (58.5)	1594 (58.5)	3132 (58.6)	0.142 ^1^
	A little, n (%)	2789 (34.6)	921 (33.8)	1868 (34.9)	
	Quite a lot, n (%)	450 (5.6)	173 (6.3)	277 (5.2)	
	Very, n (%)	107 (1.3)	39 (1.4)	68 (1.3)	
Disturbance by noise, during the day at the weekend	Not at all, n (%)	4921 (61.0)	1625 (59.4)	3296 (61.9)	0.077 ^2^
	A little, n (%)	2658 (33.0)	925 (33.8)	1733 (32.5)	
	Quite a lot, n (%)	409 (5.1)	156 (5.7)	253 (4.7)	
	Very, n (%)	74 (0.9)	29 (1.1)	45 (0.8)	
Disturbance by noise, at night	Not at all, n (%)	6083 (75.5)	1993 (72.9)	4090 (76.9)	<0.001 ^2^
	A little, n (%)	1693 (21.0)	624 (22.8)	1069 (20.1)	
	Quite a lot, n (%)	214 (2.7)	86 (3.1)	128 (2.4)	
	Very, n (%)	65 (0.8)	31 (1.1)	34 (0.6)	
Brightness of the rooms during the day	Very poor, n (%)	31 (0.4)	9 (0.3)	22 (0.4)	0.011 ^2^
	Poor, n (%)	110 (1.4)	46 (1.7)	64 (1.2)	
	Moderate, n (%)	901 (11.1)	312 (11.4)	589 (11.0)	
	Rather good, n (%)	3119 (38.4)	989 (36.0)	2130 (39.7)	
	Very good, n (%)	3952 (48.7)	1389 (50.6)	2563 (47.7)	
Disturbances by traffic	Very poor, n (%)	156 (2.0)	48 (1.8)	108 (2.1)	0.130 ^2^
	Poor, n (%)	380 (4.8)	146 (5.4)	234 (4.5)	
	Moderate, n (%)	1704 (21.4)	590 (21.8)	1114 (21.2)	
	Rather good, n (%)	2854 (35.8)	932 (34.4)	1922 (36.6)	
	Very good, n (%)	2868 (36.0)	993 (36.7)	1875 (35.7)	

N: number of participants, n: number of persons, IQR: interquartile range.^1^ Mann–Whitney U test, ^2^ Chi-squared test.

**Table 4:** Subjective evaluation of residential district.

		Total N = 8192	Without Hypertension n = 2770	Hypertension n = 5422	*p*-Value
Subjective Evaluation of Residential District					
Attractiveness of the residential district	very poor, n (%)	45 (0.6)	8 (0.3)	37 (0.7)	<0.001 ^1^
	poor, n (%)	294 (3.6)	89 (3.3)	205 (3.8)	
	moderate, n (%)	1526 (18.9)	483 (17.7)	1043 (19.6)	
	rather good, n (%)	3356 (41.6)	1087 (39.8)	2269 (42.5)	
	very good, n (%)	2846 (35.3)	1065 (39.0)	1781 (33.4)	
Shopping facilities nearby	very poor, n (%)	67 (0.8)	21 (0.8)	46 (0.9)	0.006 ^1^
	poor, n (%)	347 (4.3)	96 (3.5)	251 (4.7)	
	moderate, n (%)	1136 (14.0)	367 (13.4)	769 (14.3)	
	rather good, n (%)	3227 (39.8)	1066 (38.8)	2161 (40.3)	
	very good, n (%)	3330 (41.1)	1196 (43.6)	2134 (39.8)	
Social contacts in the neighbourhood	very poor, n (%)	66 (0.8)	24 (0.9)	42 (0.8)	0.054 ^1^
	poor, n (%)	433 (5.3)	166 (6.1)	267 (5.0)	
	moderate, n (%)	2042 (25.2)	677 (24.7)	1365 (25.5)	
	rather good, n (%)	3335 (41.2)	1085 (39.6)	2250 (42.0)	
	very good, n (%)	2221 (27.4)	785 (28.7)	1436 (26.8)	
Ambient air quality	very poor, n (%)	76 (0.9)	36 (1.3)	40 (0.7)	0.008 ^1^
	poor, n (%)	419 (5.2)	155 (5.7)	264 (4.9)	
	moderate, n (%)	1712 (21.2)	615 (22.5)	1097 (20.5)	
	rather good, n (%)	3964 (49.1)	1303 (47.6)	2661 (49.8)	
	very good, n (%)	1906 (23.6)	628 (22.9)	1278 (23.9)	
Green zones nearby	very poor, n (%)	11 (0.1)	3 (0.1)	8 (0.1)	0.192 ^1^
	poor, n (%)	82 (1.0)	29 (1.1)	53 (1.0)	
	moderate, n (%)	536 (6.6)	207 (7.5)	329 (6.1)	
	rather good, n (%)	2735 (33.7)	921 (33.5)	1814 (33.9)	
	very good, n (%)	4740 (58.5)	1586 (57.8)	3154 (58.9)	
Safety of the neighbourhood	very poor, n (%)	37 (0.5)	11 (0.4)	26 (0.5)	<0.001 ^1^
	poor, n (%)	273 (3.4)	79 (2.9)	194 (3.6)	
	moderate, n (%)	1956 (24.2)	600 (21.9)	1356 (25.4)	
	rather good, n (%)	4400 (54.5)	1511 (55.2)	2889 (54.1)	
	very good, n (%)	1413 (17.5)	538 (19.6)	875 (16.4)	

N: number of participants, n: number of persons. ^1^ Mann–Whitney U test.

**Table 5:** Results logistic (outcome is arterial hypertension) and linear (outcome is quality of life) regression analyses.

Logistic Regression	OR	2.50%	97.50%	*p*-Value
(Outcome arterial hypertension)				
EQ-5D score	0.289	0.168	0.505	<0.001
Subjective living environment index	0.998	0.976	1.008	0.468
Age in years	1.074	1.066	1.082	<0.001
Female (yes)	0.527	0.471	0.596	<0.001
ISCED = 2 (yes)	0.775	0.533	1.009	0.062
ISCED = 3 (yes)	0.566	0.388	0.740	<0.001
Smoking (yes)	0.839	0.725	0.967	0.019
Heart disease (yes)	2.686	2.179	3.122	<0.001
Linear regression, adj. R^2^ 0.07	Beta	2.50%	97.50%	*p*-value
(Outcome EQ-5D score)				
Subjective living environment index	0.005	0.004	0.006	<0.001
Arterial hypertension (yes)	−0.014	−0.021	−0.008	0.001
Age in years	0.000	−0.000	0.000	0.122
Female (yes)	−0.024	−0.031	−0.018	<0.001
ISCED = 2 (yes)	0.005	−0.010	0.020	0.476
ISCED = 3 (yes)	0.018	0.003	0.033	0.020
Smoking (yes)	−0.010	−0.019	−0.003	0.006
Heart diseases (yes)	−0.040	−0.048	−0.033	<0.001

EQ-5D: EurQol questionnaire on quality of life, ISCED: International Standard Classification of Education, OR: odds ratio.

## Data Availability

Data are available on reasonable request from the corresponding author.
